# An Exogenous NO Donor Provokes Mechanical Alternans in Normal Rat Atria and Impairs Sarcomere Contractility in Right Atrial Cardiomyocytes in Atrial Fibrillation

**DOI:** 10.3390/biom15050735

**Published:** 2025-05-17

**Authors:** Xenia Butova, Tatiana Myachina, Polina Mikhryakova, Raisa Simonova, Daniil Shchepkin, Anastasia Khokhlova

**Affiliations:** 1 Institute of Immunology and Physiology of the Ural Branch of the Russian Academy of Science, Pervomajskaya St. 106, Yekaterinburg 620078, Russia; myachina.93@mail.ru (T.M.);; 2 Department of Biomedical Engineering, Washington University, 1 Brookings Drive, St. Louis, MO 63130-4899, USA

**Keywords:** nitric oxide, NO donor, atrial fibrillation, left and right atria, cardiomyocyte contractility, mechanical alternans

## Abstract

Atrial fibrillation (AF) is the most common arrhythmia worldwide. AF is associated with a deficiency in nitric oxide (NO) production, which contributes to disturbances in the electrical and mechanical function of the atrial myocardium. NO donors are considered promising for the treatment and prevention of AF, but their effects on atrial contractility are unclear. This study examines the direct impact of a low-molecular-weight NO donor, spermine-NONOate (NOC-22), on the contractile function of atrial cardiomyocytes in paroxysmal AF. To study whether an NO donor-induced increase in NO level causes chamber-specific changes in atrial contractility, we measured sarcomere length (SL) dynamics in contracting single cardiomyocytes from the rat left and right atria (LA, RA) using a 7-day acetylcholine-CaCl_2_-induced AF model. We showed that in control rats NOC-22 provoked alternans of sarcomere shortening in both LA and RA cardiomyocytes. In AF, NOC-22 decreased the sarcomere-shortening amplitudes and velocities of sarcomere shortening–relengthening and increased the magnitude of sarcomere-shortening alternans only in RA cardiomyocytes. The negative effects of NO donors on RA contractility warrant careful consideration of their use in AF treatment.

## 1. Introduction

Nitric oxide (NO), a small gas molecule synthesized from L-arginine by the enzyme nitric oxide synthase (NOS), is a major modulator of electromechanical coupling and mechano-electric feedback in the myocardium [1,2]. NO directly via S-nitrosylation and indirectly via cGMP/PKG signaling mediates the activity of ion channels, intracellular Ca^2+^ handling, and sarcomeric proteins, and also affects NOS function itself, impacting cardiac contraction and relaxation [3,4,5,6]. In pathological conditions, NOS can deviate from its normal function, producing a superoxide anion (O_2_^−^) instead of NO. An NO deficiency and a shift from NO to superoxide production, also known as NOS uncoupling, have been observed in both short- and long-term atrial fibrillation (AF) [7,8,9,10], highlighting its potential role in AF pathogenesis.

AF is the most prevalent arrhythmia, characterized by a disruption in atrial electrical excitation and conduction, which ultimately results in atrial contractile dysfunction [11]. Low-molecular-weight NO donors are considered promising for the treatment and prevention of AF due to their multifaceted benefits [2,12,13]. By reducing oxidative stress and improving endothelial function, NO donors may counteract ROS-driven pathological signaling in AF [13,14]. NO donors can reduce fibrosis and inflammation, which contribute to the structural remodeling sustaining AF [6,15,16]. NO donors modulate various cardiac ion channels, including K^+^ and Ca^2+^ channels, which are critical for atrial electrophysiology. Previous studies have demonstrated that NO donors SNAP and DEANO prolong action potential (AP) duration in AF by decreasing transient outward K^+^ currents (I_to_) [17,18]. This modulation can help to stabilize atrial electrical activity, reduce arrhythmogenic triggers, and potentially prevent or terminate AF episodes. However, much less is known about the effects of NO donors on atrial contractility. While studies have reported negative inotropic effects of NO donors on the atrial strips from healthy hearts [19,20], the possible modulation of atrial contractility by NO donors in AF is unclear. In this study, we examine the direct effects of NO donors on the contractile activity of atrial cardiomyocytes in a paroxysmal AF model.

In our previous study, we have shown that paroxysmal AF impairs myocardial structure and sarcomere shortening in atrial cardiomyocytes predominantly in the left atrium (LA), and not in the right atrium (RA) [21]. Here, we hypothesize that an application of NO donors in AF has different effects on the LA and RA contractility. Previous studies have observed the chamber-specific differences in NO production, which may cause the different sensitivity of LA and RA to the application of NO donors. Cai et al. [7] showed that the NO levels were decreased in LA but were not altered in RA in an AF porcine model. Reilly et al. [8] demonstrated that NOS expression and activity were higher in LA than in RA in norm and significantly decreased in LA in AF. To study whether an increased NO level contributes to chamber-specific changes in contractility in AF, we measured sarcomere length (SL) dynamics in single LA and RA cardiomyocytes from rats with acetylcholine(ACh)-CaCl_2_-induced AF using a low-molecular-weight NO donor spermine-NONOate (NOC-22).

## 2. Materials and Methods

### 2.1. Ethical Approval

All procedures involving animal care and handling were performed according to the guidelines stated in Directive 2010/63/EU of the European Parliament and approved by the Animal Care and Use Committee of the Institute of Immunology and Physiology of RAS (protocol № 06/20 from 10 November 2020). Male Wistar rats at 9 weeks of age were obtained from the animal house of the Institute of Immunology and Physiology. They were randomly divided into the groups with AF and age-matched intact control rats (N > 3 in each group). Rats with AF and control rats were caged separately in groups of 5–6 per cage at 22–24 °C under a 12:12 h light–dark cycle and with unlimited access to food (Delta Feeds LbK 120 S-19, BioPro, Novosibirsk, Russia) and water. Unless otherwise noted, all chemicals and reagents were purchased from Sigma-Aldrich (St. Louis, MO, USA).

### 2.2. Experimental Model of Paroxysmal AF

Paroxysmal AF in rats was induced using the ACh-CaCl_2_-induced AF model [22] with modifications. Briefly, rats were injected intravenously with AChCl (60 μg/mL) and CaCl_2_ (10 mg/mL) at 1.7 mL/kg for 7 days. AF episodes (f-waves, invisible P-waves, and irregular R-R intervals, with a duration ≥ 30 s on ECG) were detected using a three-channel electrocardiograph (ECG300G-VET, Contec, Shanghai, China). All control rats were in sinus rhythm. Seven days after the first ACh-CaCl_2_ injection, rats were heparinized with 5000 IU/kg sodium heparin (Ellara, Pokrov, Russia), anesthetized with an intramuscular injection of 1 mL/kg Xylazine 2% (Alfasan, Woerden, The Netherlands) and 0.3 mL/kg tiletamine + zolazepam (Zoletil 100^®^, Virbac, Carros, France), and euthanized by exsanguination.

### 2.3. Atrial Cardiomyocyte Isolation

Single cardiomyocytes from LA and RA were isolated using a combined technique of Langedorff perfusion and intra-chamber injections described in detail elsewhere [23]. For measurements of NO production, cardiomyocytes were stored in a low-Ca^2+^ modified Tyrode solution (140.0 mM NaCl, 5.4 mM KCl, 1.0 mM MgSO_4_, 10.0 mM HEPES, 11.1 mM D-glucose, and 0.025 mM CaCl_2_, pH 7.35) to prevent spontaneous contractions of atrial cardiomyocytes during recordings. For measurements of sarcomere shortening, cardiomyocyte suspensions were stored in a modified Tyrode solution with 1.8 mM CaCl_2_ (140.0 mM NaCl, 5.4 mM KCl, 1.0 mM MgSO_4_, 10.0 mM HEPES, 11.1 mM D-glucose, and 1.8 mM CaCl_2_, pH 7.35) at room temperature (22 ± 2 °C). Isolated single cardiomyocytes were kept at rest for at least 30 min before being used in experiments within 4–6 h.

### 2.4. Measurements of NO Contents in Atrial Cardiomyocytes

Intracellular NO levels in atrial cardiomyocytes were visualized using 5 μM diaminofluorescein-FM diacetate (DAF-FM) and analyzed as described elsewhere [21]. 

### 2.5. Incubation of Atrial Cardiomyocytes with a NO Donor

Spermine N-(2-aminoethyl)-N-(2-hydroxy-2-nitrosohydrazino)-1,2-ethylenediamine (NOC-22, half-life of NO release = 230 min in PBS at 22 °C, pH 7.4) as a well-characterized NO donor with the consistent NO release [13,24,25] was used to study the effects of the NO donor on atrial cardiomyocyte contractility in AF. The frozen stock solution (10 mM) was diluted in a low-Ca^2+^ modified Tyrode solution to achieve a concentration of 400 μM. Before SL measurements, resting cardiomyocytes were incubated with NOC-22 in a modified Tyrode solution with 1.8 mM CaCl_2_ in an experimental chamber at 30 °C, and then were field-stimulated at 1 Hz to achieve steady-state contractions. The total time of cell incubation with NOC-22 including the time of measurements did not exceed 15 ± 1 min.

### 2.6. Measurements of SL Length and Sarcomere-Shortening Alternans in Single Atrial Cardiomyocytes

SL changes during mechanically non-loaded cardiomyocyte contractions were measured using the IonOptix system (IonOptix Corporation, Milton, MA, USA) at a pacing frequency of 1 Hz and 30 °C.

At steady-state conditions, the last 10 beats were averaged and the following characteristics of sarcomere shortening–relengthening were analyzed: end-diastolic sarcomere length (EDSL), absolute sarcomere-shortening amplitude (EDSL minus end-systolic SL), fractional sarcomere-shortening amplitude normalized by EDSL, and maximum velocities of sarcomere shortening (vshort) and relengthening (vrel). .

Alternans of sarcomere shortening were analyzed as described previously [26]. Two variations from the sarcomere-shortening amplitude higher than 5% from the not-alternating beats (before and after the appearance of alternans) were estimated and the magnitude of change was calculated..

### 2.7. Statistical Analysis

All experimental data were analyzed using R v4.0 in the R Studio environment (RStudio Team, Integrated Development for R., Boston, MA, USA) and GraphPrism 8.0 software (Origin Lab, Northampton, MA, USA). Data are expressed as median and interquartile range. The parameters of Gaussian distribution were checked by the Shapiro–Wilk test. Homogeneous dispersion between groups was tested using Bartlett’s test. The statistical comparisons were performed using the Scheirer–Ray–Hare test, followed by Dunn’s multiple comparisons test and 2-way ANOVA test with Sidak’s multiple comparisons test. A *p*-value of <0.05 was considered to indicate a significant difference between groups.

## 3. Results

### 3.1. In AF, NOC-22 Increases NO Levels More in RA Cardiomyocytes than in LA Cardiomyocytes

**Figure 1:**
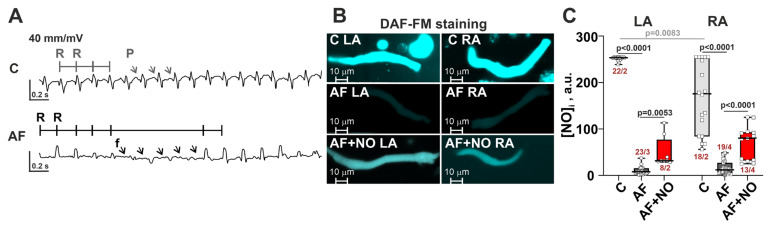
The effects of NOC-22 on intracellular NO levels in LA and RA cardiomyocytes in rats with AF. (**A**) Representative ECG recordings from control rats (C, top) and from rats with ACh-CaCl_2_-induced AF (AF, bottom). (**B**) Confocal images of cardiomyocytes stained with DAF-FM without and with NOC-22 incubation, magnification 40×/Oil. (**C**) Intracellular NO levels ([NO]_i_) in LA and RA cardiomyocytes from the control rats (**C**), rats with AF (AF), and rats with AF after 15 min incubation with NOC-22 (AF+NO). Data are presented in box and whisker plots. Each dot represents an individual cardiomyocyte. The number of n cardiomyocytes from N hearts in each group is shown as n/N. Scheirer–Ray–Hare test with Dunn’s multiple comparisons test.

Representative ECG images in control and AF rats are shown in Figure 1A. First, we verified that paroxysmal AF induces a decrease in endogenous NO production in atrial cardiomyocytes. Consistently with previous findings, AF reduced NO levels by ~97% and ~93% in LA and RA cardiomyocytes, respectively [21]. Short-time (15 min) incubation of cardiomyocytes from the AF group with NOC-22 led to an increase in intracellular NO content by ~13% in LA, and by ~45% in RA (Figure 1B,C).

### 3.2. In AF, NOC-22 Reduces the Amplitude and Velocities of Sarcomere Shortening and Relengthening Only in RA Cardiomyocytes

**Figure 2:**
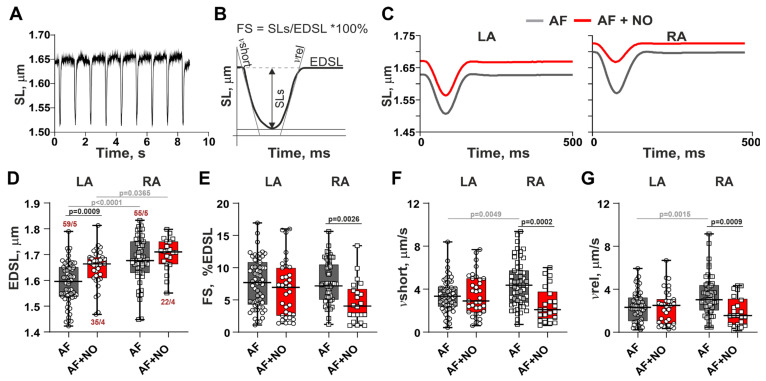
The effects of NOC-22 on sarcomere shortening in LA and RA cardiomyocytes in rats with AF. (**A**) Representative recordings of sarcomere length (SL) changes during cardiomyocyte contractions. (**B**) Characteristics of SL changes derived from (**A**). (**C**) Representative SL changes in contracting LA and RA cardiomyocytes from the AF rats before and after NOC-22 incubation. (**D**–**G**) The effects of NOC-22 on the end-diastolic SL (EDSL), fractional sarcomere-shortening amplitude normalized by EDSL (FS), maximum velocity of sarcomere shortening (vshort), and maximum velocity of sarcomere relengthening (vrel). Each dot represents an individual cardiomyocyte. The number of n cardiomyocytes from N hearts in each group is shown as n/N, 2-way ANOVA test with Sidak’s multiple comparisons test.

Representative recordings of SL changes during cardiomyocyte contractions and the derived characteristics are shown in Figure 2A,B. Representative traces of steady-state sarcomere shortening–relengthening in cardiomyocytes from AF rats before and after NOC-22 incubation are shown in Figure 2C. An application of NOC-22 to LA cardiomyocytes from AF rats increased EDSL (Figure 2D), but did not affect the amplitude and velocity of sarcomere shortening (Figure 2E–G). In RA cardiomyocytes, NOC-22 did not change EDSL but decreased the sarcomere-shortening amplitude by ~16% and reduced velocities of sarcomere shortening and relengthening by ~25% and ~8%, respectively. The LA vs. RA differences in EDSL, and velocities of sarcomere shortening and relengthening observed in AF were eliminated after the application of NOC-22.

### 3.3. In AF, NOC-22 Enhances Sarcomere-Shortening Alternans in RA Cardiomyocytes

**Figure 3:**
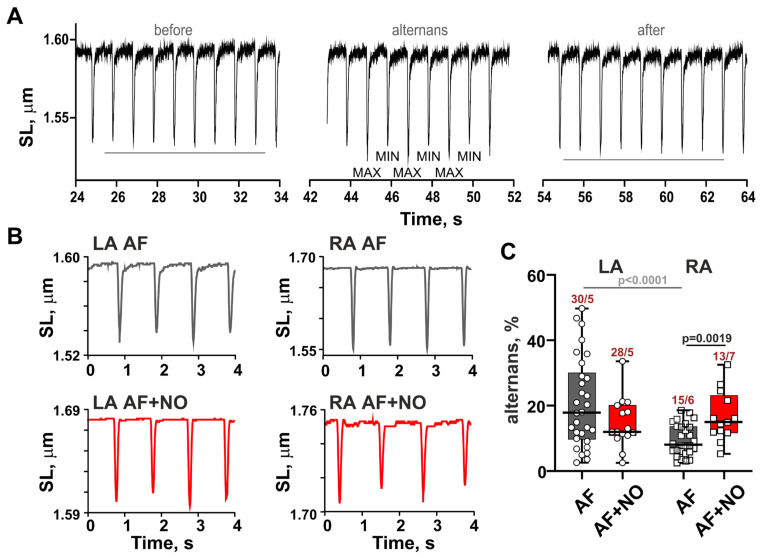
The effects of NOC-22 on sarcomere-shortening alternans in rats with AF. (**A**) A representative sarcomere-shortening recording with an episode of alternans when MIN and MAX variations of the sarcomere-shortening amplitude before and after the appearance of alternans were estimated and the magnitude of change was calculated. (**B**) Representative recordings of alternans in LA and RA cardiomyocytes from AF rats before and after NOC-22 incubation. (**C**) The effects of NOC-22 on the alternans magnitudes in AF. Data are presented in box and whisker plots. Each dot represents an individual cardiomyocyte. The number of n cardiomyocytes from N hearts in each group is shown as n/N, 2-way ANOVA test with Sidak’s multiple comparisons test.

Representative recordings of sarcomere-shortening alternans are shown in Figure 3A. Consistent with our previous results [26], AF caused an appearance of sarcomere-shortening alternans (Figure 3B) in 51% of cases for both LA and RA cardiomyocytes. An addition of NOC-22 did not change the alternans’ appearance but increased the alternans’ magnitude only in RA cardiomyocytes by ~85% (Figure 3C). While in LA cardiomyocytes we observed ~55%-greater magnitudes of alternans compared to RA cardiomyocytes, NOC-22 eliminated these inter-atrial differences.

Thus, in AF, LA and RA cardiomyocytes have different sensitivity to NOC-22. In RA cardiomyocytes, an incubation with NOC-22 reduces the amplitude and velocities of sarcomere shortening–relengthening and increases the magnitude of shortening alternans. In LA cardiomyocytes, NOC-22 almost has no effects on the sarcomere shortening, only increasing end-diastolic sarcomere length.

### 3.4. In Norm, NOC-22 Provokes Sarcomere-Shortening Alternans in LA and RA Cardiomyocytes

**Figure 4:**
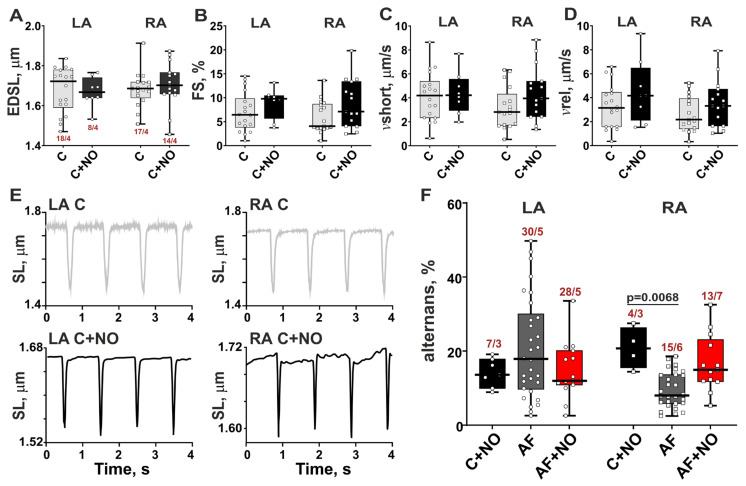
The effects of NOC-22 on sarcomere shortening in LA and RA cardiomyocytes in control (intact) rat hearts. (**A**) End-diastolic sarcomere length (EDSL), (**B**) fractional sarcomere-shortening amplitude normalized by EDSL (FS), (**C**) maximum velocity of sarcomere shortening (vshort), and (**D**) maximum velocity of sarcomere relengthening (vrel). (**E**) Representative recordings of sarcomere-shortening alternans induced by NOC-22 in atrial cardiomyocytes from control rats. Macro SS alternans are shown in boxes. (**F**) The comparison of NOC-22 effects on magnitudes of alternans in control and AF groups. Data are presented in box and whisker plots. Each dot represents an individual cardiomyocyte. The number of n cardiomyocytes from N hearts in each group is shown as n/N, 2-way ANOVA test with Sidak’s multiple comparisons test.

An addition of NOC-22 to LA and RA cardiomyocytes from control rats showed no significant impact on the parameters of SL dynamics (Figure 4A–D). However, NOC-22 induced shortening alternans in 40% of LA cardiomyocytes and 17% of RA cardiomyocytes (Figure 4E,F). In LA cardiomyocytes from the C+NO group, the magnitude of alternans was 14%, which is comparable with the magnitudes in the AF and AF+NO groups. In RA cardiomyocytes from the C+NO group, the magnitude of alternans was 21%, which is higher compared to the AF group (Figure 4F).

Thus, in normal hearts, NOC-22 destabilizes sarcomere shortening in single LA and RA cardiomyocytes and does not affect the amplitude and velocity of SL dynamics.

## 4. Discussion

To our knowledge, we show for the first time the direct effects of NO donors on atrial contractility in AF. The main findings of this study are as follows: (i) in paroxysmal AF, NOC-22 impaired SL dynamics and increased the magnitude of sarcomere-shortening alternans in RA cardiomyocytes but not in LA cardiomyocytes; (ii) in norm, NOC-22 caused the appearance of alternans with a magnitude comparable to that observed in AF for LA cardiomyocytes, and with a magnitude ~2-fold greater for RA cardiomyocytes.

LA and RA have distinct features in their structure and functional activity that contribute to their different roles in the cardiac cycle and the maintenance of AF [8,27]. In our previous study, we showed that ACh-CaCl_2_-induced paroxysmal AF led to a chamber-specific response in rat atrial cardiomyocytes, decreasing the sarcomere-shortening amplitudes and the velocity of sarcomere relengthening in LA cardiomyocytes, and increasing end-diastolic sarcomere length and the velocity of sarcomere shortening in RA cardiomyocytes compared to control rats [21]. An ACh-CaCl_2_-induced AF model recapitulates the arrhythmogenic phenotype of AF due to a decrease in the atrial effective refractory period, which creates a substrate for AF via the activation of ACh-activated K^+^ channels (I_KACh_) and hypercalcemia [22,28].

An imbalance in NO production plays a central role in the development and maintenance of AF [15,29,30,31]. Consistent with our previous work [21] and literature data [7,8,9], AF is characterized by a deficiency in intracellular NO levels in atrial myocardium. NO affects ionic currents, Ca^2+^ handling, and sarcomere protein phosphorylation in atrial cardiomyocytes [1,15,32,33]. Incubation of isolated atrial cardiomyocytes with the NO donor SIN-1 delayed AP shortening during short-term rapid atrial pacing, as well as increased L-type Ca^2+^ current (I_CaL_) and the amplitude of intracellular [Ca^2+^]_i_ transients in normal sheep hearts [17]. In human chronic AF, NO donors prolonged AP duration by decreasing IKv4.3 and I_to1_ in LA cardiomyocytes [18]. The authors concluded that cGMP-dependent NO signaling is disrupted in AF possibly because of the attenuated sGC-cGMP-inhibited phosphodiesterase (PDE3) interaction [34]. The application of the NO donors SNAP and DEA-NONOate reduced the force of contraction of atrial preparations in healthy mice [19]. Studies have shown that NO-stimulated cGMP modulates cardiac contractility and enhances relaxation kinetics via PKG-dependent phosphorylation of L-type Ca^2+^ channels, titin, TnI, and phospholamban [4,5].

Here we demonstrated that a low-molecular-weight NO donor spermine-NONOate (NOC-22) reduced the sarcomere-shortening amplitude and velocities of sarcomere shortening–relengthening, as well as increased the magnitude of sarcomere-shortening alternans in RA cardiomyocytes but not in LA cardiomyocytes from rats with paroxysmal AF. The negative inotropic response of RA cardiomyocytes to a NO donor might be caused by a not fully compensated NO/redox balance. Previously, we have shown that a deficiency in intracellular NO levels is accompanied with an increased ROS production in ACh-CaCl_2_-induced paroxysmal AF [21]. An increase in ROS production is linked to the initiation, development, and maintenance of AF [35,36,37].

A short-term incubation of atrial cardiomyocytes with NOC-22 partially restored intracellular NO production with a greater extent in RA cardiomyocytes than in LA cardiomyocytes. Previous studies showed that NO/redox imbalance in AF is caused not only by a decrease in NO release [7,8,9,10], but also by changes in NOS expression and activity, as well as a deficiency of tetrahydrobiopterin (BH_4_) for NO synthesis [29,38]. Whether the impaired NO/redox imbalance is involved in the chamber-specific response of atrial cardiomyocytes to NO donors requires further studies.

Consistently with our previous findings [21], we did not find LA vs. RA differences in the parameters of SL dynamics in control rats, while AF provoked the inter-atrial differences in end-diastolic sarcomere length as well as in velocities of sarcomere shortening–relengthening. An addition of NOC-22 to atrial cardiomyocytes from rats with AF restored the inter-atrial homogeneity in SL dynamics but significantly attenuated sarcomere shortening in RA cardiomyocytes.

The mechanical alternans underlie arrhythmia formation and sustainability [39,40]. In AF, we observed alternans of sarcomere shortening in both LA and RA cardiomyocytes, with a greater magnitude in LA cardiomyocytes than in RA cardiomyocytes. We found that NOC-22 increased the magnitudes of sarcomere-shortening alternans in RA cardiomyocytes in AF and provoked alternans activity in both LA and RA cardiomyocytes in normal hearts. We may suggest that NO donors can promote an arrhythmogenic substrate that sustains AF, particularly in the RA myocardium. The mechanisms of NO-mediated cardiac alternans have been only defined for ventricular myocardium. In healthy mouse ventricles, Power et al. [2] showed that NO-mediated S-nitrosylation of CaMKIIδ at cysteine-290 increased Ca^2+^ spark frequency, sustaining β-adrenergic receptor-induced arrhythmias. The mechanisms of mechanical alternans in atrial myocardium induced by NO donors remain to be elucidated.

## 5. Conclusions

In this study, we estimated the chamber-specific changes in rat LA and RA contractility in AF in response to a NO donor spermine-NONOate (NOC-22). In AF, NOC-22 impaired the sarcomere contractility only in RA cardiomyocytes. In norm, NOC-22 provoked mechanical alternans in both LA and RA cardiomyocytes. We propose that NO donors may contribute to the development of an arrhythmogenic substrate that sustains AF, especially in the RA myocardium. The use of NO donors and NO-releasing drugs in vivo requires careful consideration due to their potential negative effects on sarcomere contractility and the different sensitivity of LA and RA to an increase in NO levels. Further research into the molecular mechanisms could provide valuable insights into the inter-atrial sensitivity to NO donors in AF and potentially lead to more targeted therapeutic approaches.

## 6. Limitation

The AF model we used does not account for all the underlying causes of AF (such as post-operative AF, etc.). However, animal models remain valuable tools for investigating specific hypotheses about drug-related effects and for identifying mechanisms that can be further explored in human studies. The results of this study must be interpreted with caution since the effects of acute application of NO donors are likely to depend on the NO donor type, concentration range, and the incubation time, and differ from chronic treatment with NO donors. Further washout experiments could be helpful to determine whether the acute effects of NOC-22 are reversible and if the observed contractile changes are temporary.

## Data Availability

All experimental data generated or analyzed during this study are included in this article.

## Abbreviations

The following abbreviations are used in this manuscript:

ACh	Acetylcholine
AF	Atrial fibrillation
BH_4_	tetrahydrobiopterin
CaMKIIδ	Calcium calmodulin dependent protein kinase II
cGMP	Cyclic guanosine monophosphate
DAF-FM	Diaminofluorescein-FM diacetate
EDSL	End-diastolic sarcomere length
FS	Fractional sarcomere-shortening amplitude normalized by EDSL
LA	Left atrium
NO	Nitric oxide
NOC-22	Spermine-NONOate
NOS	Nitric oxide synthase
PKG	cGMP-dependent protein kinase
RA	Right atrium
ROS	Reactive oxygen species
SL	Sarcomere length
vrel	Maximal velocity of sarcomere relengthening
vshort	Maximal velocity of sarcomere shortening
